# A scoping review of the spatial perception of tinnitus and a guideline for the minimum reporting of tinnitus location

**DOI:** 10.1080/03036758.2024.2344781

**Published:** 2024-05-01

**Authors:** Grant D. Searchfield, Philip J. Sanders, Amit Barde

**Affiliations:** a School of Population Health, Faculty of medical and Health Sciences, The University of Auckland, Auckland, New Zealand; b Eisdell Moore Centre, School of Population Health, Faculty of medical and Health Sciences, The University of Auckland, Auckland, New Zealand; c TrueSilence Therapeutics Inc. Atlanta, Georgia, USA

**Keywords:** Tinnitus, spatial, localisation, lateralisation, mechanisms, therapy, treatment, guideline

## Abstract

Tinnitus spatial localisation is an essential attribute of tinnitus perception and how it is separated from other ongoing neural activity. A scoping review was undertaken to determine how tinnitus localisation is reported, the role of the perceived spatial location of tinnitus on neurophysiology and if sound presented spatially can change tinnitus perception. Following reading of the full-text articles and including articles from reference lists, 46 articles were included for review. Six themes emerged from the results. 1. Where tinnitus was localised. 2. The effects of tinnitus on localisation. 3. The mechanisms underpinning tinnitus spatial location. 4. Masking. 5. Auditory training. 6. Multisensory training and virtual reality (VR). Tinnitus is much more complex than the often-used description of ‘ringing in the ears’. Tinnitus can be heard anywhere in and around the head. Spatial sound presentation and perceptual training approaches may disrupt spatial selective attention to tinnitus and appear as changes in some of the neural networks involved in sound localisation. Where tinnitus is heard is a critical aspect of its perception, but its report, even in studies purporting to study localisation, is too general. A matrix for standardised minimum reporting of tinnitus location is recommended.

## Introduction

Spatial hearing is essential for our survival as we navigate through our acoustic environment (Avan et al. 2015). Localisation of sound enables focus on targets while ignoring distractors. The authors have observed that patients and research participants commonly search for the source of the sound when hearing tinnitus for the first time. True sounds have a physical sound source, an auditory object creating them, while subjective tinnitus does not Searchfield 2014). The disconnect between tinnitus and the environment around us, creates an unreal, difficult to ignore perception (Feldmann 1992).

The localisation of true sound utilises three physical characteristics of sound waves interacting with the head and ears – two binaural cue types and one spectral. The binaural localisation cues are Interaural Time Difference and Interaural Level Difference. The spectral cue is monaural and, as the name implies, frequency based (Bloom 1977). Together, these cues make up the Head Related Transfer Function (HRTF) (Cheng and Wakefield 1999; Grothe et al. 2010). As well as identifying where a sound is coming from, spatial cues are also used to aid the separation of target sounds of interest from background distracting ‘noise’ (Kidd et al. 1998; Avan et al. 2015). Hearing loss can disrupt this spatial hearing resulting in neuro-plastic changes that can change with time and experience (Avan et al. 2015). Due to the known association between hearing acuity and tinnitus, it follows that impaired spatial perception, reactive and experienced-based auditory spatial plasticity will accompany tinnitus too. Neural network plasticity in response to changes in binaural spontaneous cochlear-neural activity is a likely driver in where tinnitus is perceived (De Ridder et al. 2022). It has also been proposed that understanding how tinnitus is localised in space, and how this information is extracted from sound driven and other endogenous neural activity is critical to the development of tinnitus treatments (Searchfield 2014).

We undertook a scoping review to catalogue and thematically evaluate the current understanding of the spatial perception of tinnitus. Key concepts in the research of tinnitus spatial perception/localisation were mapped and reviewed, without applying strict exclusion criteria applied in systematic reviews (Arksey and O'Malley 2005). Scoping reviews are particularly valuable in new or complex areas of research, such as tinnitus localisation, in which there is presumed to be limited depth of knowledge.

## Methods

**Figure 1.**
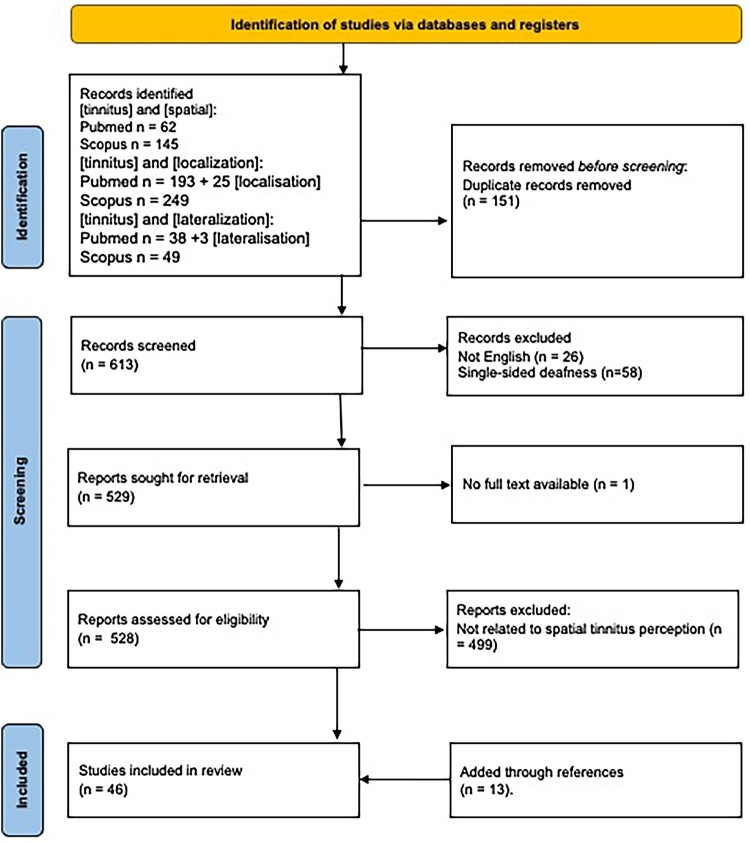
PRISMA flowchart for database searching and study inclusion.

A review of the literature was undertaken using a scoping review framework (Arksey and O'Malley 2005). The research question was ‘how does the spatial location of tinnitus affect its perception, mechanisms and management?’. To identify relevant studies, database searches of Scopus (keywords) and PubMed (title and abstract) were carried out in March 2024 using the following search terms [tinnitus] and [spatial]; [tinnitus] and [localisation]; [tinnitus] and [lateralisation]; for PubMed a search using United Kingdom English spelling [localisation] and [lateralisation] returned additional unique articles (PRISMA diagram, Figure 1). After excluding duplicates 613 articles were screened and 26 were excluded for not being in English text and 57 were excluded due to focus on single-sided deafness (SSD). Of the 530 papers, 1 was not available in full text, 502 were not deemed relevant in addressing the spatial perception of tinnitus (e.g. were about objective tinnitus or anatomical ‘location’ especially neuroanatomy, unrelated to spatial perception of tinnitus). The reference list of papers and citations of included papers were searched for additional citations leading to the addition of 25 articles and 1 database. In total 46 articles were arranged into themes. The thematic grouping was undertaken by author GDS, and the themes were checked by AB and PJS. The publications were grouped according to the following themes: localisation of tinnitus, tinnitus’ effect on localisation of sound, mechanisms of tinnitus localisation, sound therapy (masking and cochlear implants), auditory training, multisensory training and VR. The narrative for each theme was assembled (supplementary Table 1) along with summary information (author, year, method, number of participants, comments). After thematic grouping a model summarising the literature was created.

## Results

### Tinnitus localisation

This theme was comprised of data from 7 articles (Axelsson and Ringdahl 1989; Stouffer and Tyler 1990; Erlandsson et al. 1992; Lim et al. 2010; Bertet et al. 2013; Searchfield et al. 2015; Cuesta and Cobo 2021) and a database (Meikle et al. 2004) (Supplementary Table 1). Surveys and clinical databases indicate that tinnitus can be heard in the ears, head, and a combination of places. Most tinnitus sufferers have tinnitus localised to both ears, but when heard in one, tinnitus is more likely in the left ear (Axelsson and Ringdahl 1989; Stouffer and Tyler 1990;Meikle et al. 2004; Lim et al. 2010). A summary of the spatial location of primary / worst tinnitus illustrating the variation in tinnitus location categories is shown in Table 1. In response to the question ‘where does your predominant tinnitus sound appear to be coming from?’ patients attending the Oregon Tinnitus clinic reported a wide range of spatial locations (Meikle et al. 2004) (Table 2). The tinnitus data archive has data concerning localisation changes since tinnitus onset. Most (85%) patients reported no change in the tinnitus location, a minority (10%) reported unilateral tinnitus that became bilateral over time (Meikle et al. 2004).

**Table 1.** Summary of tinnitus localisation characteristic from 4 surveys. Percentage scores are reported to nearest whole %. Studies used different categories ([-] data not collected).

Localisation	Authors					
	Axelsson & Ringdahl	Stouffer & Tyler	Meikle et al.	Lim et al.	Cuesta & Cobo	Erlandsson et al
Left ear/side	20	21	40	34	35	38
Right ear/side	13	16	32	22	16	30
Both ears/sides	40	52	17	37	49	-
Within head	9	10	8	4	-	-
Both ears and within head	9	-	-	-	-	-
Right ear and within head	4	-	-	-	-	-
Left ear and within head	2	-	-	-	-	-
Both ears or one and within head	-	-	1	-	-	-
outside head	-	1	-	-	-	-
Missing/unsure/variable	1	-	2	3	-	-
Other	-	-	1	-	-	32

**Table 2.** Detailed breakdown of tinnitus spatial perception recorded in the Oregon tinnitus data archive (Meikle et al. 2004).

Spatial location	*N*	% of 1629
Both ears	1026	63
Left ear	212	13
Inside head–left	91	5.6
Outside head–left	9	0.6
Not sure–left	2	0.1
Right ear	176	10.8
Inside head–right	80	4.9
Outside head–right	11	0.7
Not sure–right	-	-
Fills head	186	11.4
Inside top of head	22	1.4
Outside top of head	3	0.2
Surrounds head	5	0.3
Back of head	49	3
Other answer	96	5.9

Participants could report more than one response.

The use of external sounds to match tinnitus location in space has been investigated using sounds presented with different interaural levels. Patients controlled the level of an auditory replica of their tinnitus to lateralise their tinnitus. The level change required for a shift from the ipsilateral to the contralateral side was on average 29 dB (Bertet et al. 2013). Using an average HRTF tinnitus has been matched in the horizontal and vertical plane (Searchfield et al. 2015). Participants could localise sound to a position in or around the head that was a good match to their self-report of location. The study showed that when repeated 1 week later the spatial matching method had similar test-retest reliability to pitch and loudness matching (Searchfield et al. 2015).

### The effect of tinnitus on localisation of sound

All seven studies catalogued (Schmielau 2009; Niewiarowiczw and Kaczmarek 2011; An et al. 2012; Hyvärinen et al. 2016; Elsherif et al. 2021; Long et al. 2023a, 2023b) identified that tinnitus interfered with sound localisation in some way (Supplementary Table 1). Sound localisation was poorer when sound was presented to the ear with predominant tinnitus (An et al. 2012). In a study of vertical plane localisation, there was no difference between groups in a binaural listening condition, but in monaural listening the tinnitus group localised significantly worse with the tinnitus ear (Hyvärinen et al. 2016).

### Mechanisms of tinnitus localisation

Fifteen studies were catalogued as primarily informing mechanisms of tinnitus (Supplementary Table 1). Behavioural conditioning methods have enabled experimental measurement of tinnitus-like responses in animals. Heffner and Koay (2005) and Heffner (2011) trained rats to respond to left and right sounds. A shift in responding during silence to side of the noise exposed ear indicated that the animals were spatially localising tinnitus to that ear. In hamsters, choline acetyltransferase activity increased after intense tone exposure on the tone-exposed side as compared to the opposite side (Jin et al. 2006). Two months after exposure, choline acetyltransferase activity was still higher in the ipsilateral DCN.

In a sample of 1033 clinic patients with data on the Oregon Health Sciences Tinnitus Data archive, left-sided tinnitus appeared to be related to experience shooting guns (right-handed shooters are more likely to have left-sided hearing loss), but there was a weak association with degree of hearing loss (Meikle and Griest 1992). Tinnitus was associated with hearing asymmetry in 833 adults (Genitsaridi et al. 2021) and nonlateralised tinnitus was less likely to be associated with hearing asymmetry (Shin et al. 2023). The maximum interaural difference most strongly discriminated unilateral from bilateral tinnitus (Genitsaridi et al. 2021). An interaural threshold difference of 15 dB predicted tinnitus laterality (Tsai et al. 2012). In a sample of 62 participants those with a greater hearing loss on the right side reported right-sided tinnitus, while the majority of subjects with a greater loss in the left ear reported tinnitus heard identically in both ears or tinnitus heard louder on the right side (Cahani et al. 1984). It was speculated that this difference was related to differential hemispheric responsiveness to abnormal neural activity following peripheral damage (Cahani et al. 1984). Tinnitus in the better hearing ear can occur. In a small sample (two groups of 22) tinnitus in the better ear was associated with earlier onset hearing loss; experience may be required for tinnitus to localise to worst hearing ear (Lee et al. 2021).

Whether tinnitus is primarily represented ipsilaterally, contralaterally to tinnitus, or is represented primarily in one hemisphere has been investigated. Early evidence came from Positron Emission Tomography (PET) studies in the late 1990s. In eleven patients with tinnitus of varying spatial description 9 had increased metabolic activity in the left, 1 in the right primary auditory cortex (Arnold et al. 1996). Four participants who could alter tinnitus with oral facial movements identified activation of the auditory cortex contralateral to the ear in which tinnitus was perceived (Lockwood et al. 1998). In a magnetoencephalography study cortical maps were distorted from normal similarly ipsilateral and contralateral to the tinnitus ear (Mühlnickel et al. 1998).

Using functional Magnetic Resonant Imaging (fMRI) two independent studies using similar methods (Melcher et al. 2000; Smits et al. 2007) demonstrated, that in response to sound, there was a low percent signal change in the inferior colliculus contralateral, but not ipsilateral. Interpretation was complicated by the possibility that the sound used to obtain the fMRI suppressed ipsilateral IC activity, creating asymmetry. Another fMRI study found connectivity patterns between auditory nuclei and the lateralisation of the sound-evoked responses did not relate to the laterality of tinnitus (6 participants right-sided tinnitus, 8 left-sided tinnitus) (Lanting et al. 2014). The data of 194 patients receiving low-frequency transcranial magnetic stimulation over the left temporal cortex found suppression for left-sided (ipsilateral) and bilateral tinnitus (Frank et al. 2010). Right-sided tinnitus was not suppressed with left temporal cortex stimulation. In cases of somatosensory tinnitus, its perceived location was ipsilateral to a known craniocervical injury or disorder (Levine 1999). An anatomical MRI study found that left-sided tinnitus was associated with more severe and widespread alterations in White Matter volume and White Matter microstructure than a right-sided tinnitus group (Chen et al. 2022). It is also not just auditory areas affected by tinnitus spatial perception. A tinnitus localisation network consisting of the auditory cortex, angular gyrus, parahippocampal area and superior premotor cortex has been identified using EEG (Vanneste Sven et al. 2011b). Gamma-band is increased in the parahippocampal area contralateral to the tinnitus activity while auditory cortex gamma-band activity is the same (Vanneste S. et al. 2011a). Greater electroencephalography (EEG) absolute power was found with non-lateralised tinnitus (Shin et al. 2023).

### Masking

In a sample of 89 USA military veterans, responders to masking or Tinnitus Retraining Therapy (TRT) were more likely to report tinnitus in the head as opposed to in the ears (Theodoroff et al. 2014). Six studies (Tyler and Conrad-Armes 1984; Johnson and Hughes 1992; Tyler and Stouffer 1992; Searchfield et al. 2016; Durai et al. 2021; Kubota et al. 2022) considered the effectiveness of maskers (sounds that cover tinnitus) presented at different spatial locations relative to tinnitus (Supplementary Table 1). Tyler and Stouffer (1992) compared uncorrelated and correlated binaural maskers. Correlated maskers with a time delay resulted in a considerable reduction in MMLs for some participants. Searchfield et al. (2016) manipulated masker location using HRTF functions in three studies. Study 1 was a laboratory study that compared customised spatial masking to conventional bilateral masking. Most participants (63%) reported the 3D masker as the most effective masker and Minimum Masking Levels (MML) were lower for most participants. Monaural maskers were the least preferred (5% left, 5% right). Study 2 was a 4-week cross-over field trial that compared spatial (3D) masking to a bilaterally equal (central) masker using iPods connected to hearing aids. The greatest change in narrow band MML following the spatial masker was 16 dB SL, compared with 12 dB SL after the use of the centre masker. There was an 8-point difference in Tinnitus Handicap Inventory (THI) scores between maskers; the spatial masker showing improvement while the centre masker did not. Study 3 was a 4-month crossover pilot study and found that prototype spatial hearing aid-based maskers (for 2 months) achieved equivalent results to TRT (for 2 months). Across the three studies participants had a preference for spatial masking (Searchfield et al. 2016). In a case series using various masker types, a spatial masker was effective for most (60%) participants (Durai et al. 2021). A spiking neural network was used to visualise EEG connectivity between brain regions. In responders to masking, there were large changes in the strength of connectivity centred around parietal-occipital regions (Durai et al. 2021).

Not all the research catalogued found that overlapping the spatial position of tinnitus with sound was the most effective position. Johnson and Hughes (1992) compared diotic with dichotic masking; results showed similar effects for diotic masking (perceived in the centre of the head) and dichotic masking (localised to the ears). Kubota et al. (2022) found noise presented from a distant speaker and the outside-head noise masked a simulated tinnitus (a 4 kHz tone) more effectively than noise in the ear and the inside-head noise. They suggested this was because of external focus towards the noise resulting in less attention to the internal perception of tinnitus (Kubota et al. 2022).

### Auditory perceptual training

Four studies from the same research group (Searchfield et al. 2007; Wise et al. 2015; Wise et al. 2016; Searchfield and Sanders 2022) attempted to modify tinnitus using effortful listening to spatial sound in the form of perceptual training tasks (Supplementary Table 1). Searchfield et al. (2007) attempted to reduce an individuals’ tinnitus by improving the ability to attend to natural sounds. They evaluated the effects of a 15-day (30 min/day) take-home auditory training programme using sounds presented to the left, right or centre while participants treated the tinnitus as a distraction. MMLs were reduced for 60% of participants and a correlation was found between changes in MMLs and improvements in the ability to shift attention. A game-based perceptual training method was tested in a feasibility study (Wise et al. 2015) and then clinical trial (Wise et al. 2016). The perceptual training required localisation of, and selective attention to, target sounds in the game while ignoring distracting sounds customised to the individual’s tinnitus perception. In the clinical trial, the training game resulted in significant reductions in the Tinnitus Functional Index (TFI), THI, the ability to ignore tinnitus, and annoyance rating scales compared to a visual control based on the game ‘Tetris’. The effects were sustained for at least 3 weeks following the end of training. The reductions in the TFI were correlated with improvements in a mixed auditory–visual attention task and reduction in N1 auditory-evoked potential latency to stimuli remote from tinnitus pitch (Wise et al. 2016). Wise et al. (2016) hypothesised that attention training reduced focus on tinnitus, potentially through improved selective attention to target sounds and their location.

Searchfield and Sanders (2022) compared a digital therapy providing passive sound therapy and active game-based sound therapy to an app providing passive sound therapy only over a 12-week trial. The spatial task was an automated version of the location task developed by Searchfield et al. (2007). The average change in the TFI score with the therapy was 18 points while the change in passive sound therapy control was 10 points. The number of participants with clinically meaningful change was greater with the therapy that included attention training (Searchfield and Sanders 2022).

### Multisensory training and virtual reality

Six studies were identified (Supplementary Table 1). One study employed a visual training task (Bonnet et al. 2022), 2 evaluated perceptual training using a combination of visual and tactile stimuli synchronised with the spatial representation of sound (Spiegel et al. 2015; Searchfield et al. 2021) and 3 used virtual environments with tinnitus avatars (virtual reality spatial representations of tinnitus) (Malinvaud et al. 2016; Park et al. 2022; Draper et al. 2023).

The creation of an optical deviation (prism adaptation) towards the unaffected side in disorders with unilateral symptoms has been investigated in several sensory disorders (e.g. pain). It was hypothesised that prism adaptation towards the ear contralateral to tinnitus would reduce tinnitus. In a case study, the perceived position of tinnitus was moved towards the contralateral ear (Bonnet et al. 2022). Spiegel et al. (2015) found that training using left and right audio with simultaneous visual and somatosensory stimulation could reduce unilateral tinnitus. Twenty days of training resulted in small but statistically significant reductions in TFI and Tinnitus Severity Numeric Scale scores and improved attentional abilities. The TFI reduction was still present 3 weeks following the end of the training. In a follow-up study, Searchfield et al. (2021) attempted to augment this training by combining the training with fluoxetine (to facilitate neuroplasticity). The fluoxetine provided no additional benefit, and training-induced effects were not as great as seen in the original study. Evaluation with fMRI suggested that the small changes seen with training were associated with changes in sensory and attention networks (Searchfield et al. 2021).

Malinvaud et al. (2016) described the testing of VR tasks for tinnitus, The training consisted of 1 session a week for 8 weeks, in which patients had to manipulate a copy of their tinnitus in space. The therapy’s goal was to integrate visual, auditory, and proprioceptive information with tinnitus. A control group received group Cognitive Behavioural Therapy (CBT) for 8 sessions spread over 12 weeks. Both treatments were beneficial. It was concluded that VR outcomes were broadly equivalent to the CBT (Malinvaud et al. 2016). Park et al. (2022) used a VR environment comprised of four different settings (a bedroom, a living room, a restaurant, and a city street). Participants moved their tinnitus avatar to a tinnitus disposal ‘trash’ site in the virtual environment. The tinnitus disposal sites were noisy areas on the scene. The goal was to create a cognitive illusion of absorbing the tinnitus sounds into louder environmental noises. The THI was improved by 6 points and EEG activity increased at the orbitofrontal cortex (Park et al. 2022). Draper et al. (2023) described the development of a VR treatment in which a masker sound was associated with a visual virtual object (a radio) that could be moved around in space. Three virtual environments (blank, beach and forest) were compared. The beach scene was perceived as the most relaxing, but the forest scene was preferred for tinnitus overall. It was hypothesised that many users don’t want to use VR to relax, and that the forest world was larger and offered greater opportunity for exploration of the virtual world, consequently a greater distraction from the tinnitus (Draper et al. 2023).

## Discussion

A scoping review of the literature investigated the research question: ‘how does the spatial location of tinnitus affect its perception, mechanisms and management?’. The review identified 6 themes: 1. Tinnitus localisation. 2. Effect of tinnitus on localisation. 3. Mechanisms of tinnitus localisation. 4. Masking. 5. Auditory training. 6. Multisensory training & Virtual Reality that are summarised in a visual model (Figure 1).

The overarching theme was the concept of localisation of tinnitus experience, there was consensus that tinnitus can be heard from different places in and around the head. Tinnitus is an internal, or less commonly, a peripersonal (near field) experience. A common lay description of tinnitus is ‘ringing in the ears’. The literature summarised in this review clearly demonstrates that ringing in the ears is an oversimplification. In 4 of 6 surveys, tinnitus was heard in the head most frequently, followed by the left ear. A basic question for future research is: ‘are current survey and case history categories of tinnitus location fit for purpose?’. Tinnitus location matching studies have reported that tinnitus spatial locations are more nuanced than just bilateral, unilateral or in the head (Searchfield et al. 2015). But what resolution in the description is useful? Data acquired for big datasets may not currently include the reported location of tinnitus, or in insufficient detail for future application. Given the ecological importance of spatial perception, and the arguments made here about the importance of tinnitus location, it may become important to report tinnitus location with a high degree of resolution. aSpatial matching should perhaps be included alongside tinnitus pitch and loudness matching in tpsychoacoustic tinnits assessment.

The absence of standardised reporting of the spatial location of tinnitus has ramifications to all the subthemes in the model. Comparing studies and ascertaining common neurophysiological mechanisms is precarious. Localisation is so poorly defined in neurophysiological studies that the precision of modern imaging and EEG could be considered irrelevant, i.e ‘unilateral tinnitus’ is a gross classification that could encompass many of the descriptors used by patients in more detailed classifications (Meikle et al. 2004). Mechanistic studies need to be more precise in descriptions of tinnitus location. Searchfield et al. (2015) were able to demonstrate that a reliable tinnitus localisation matching was possible, and tinnitus Avatars have been created in virtual environments that replicate tinnitus.

The lateralisation of tinnitus was most often associated with poorer hearing ear (Genitsaridi et al. 2021). The higher proportion of left-sided tinnitus found in most studies (Axelsson and Ringdahl 1989; Stouffer and Tyler 1990; Meikle et al. 2004; Lim et al. 2010) has been attributed to shooting (for example in communities with hunting traditions or military service) (Meikle and Griest 1992). The left ears of right-handed shooters are exposed to higher levels of sound and greater injury (Meinke et al. 2017). However, counter to this explanation for left-sided tinnitus, Meikle and Griest (1992) did not find significant differences in audiometric thresholds between ears. It is now accepted that the audiogram may under-represent hearing damage complicating the interpretation of the initial source of tinnitus (Plack et al. 2014). Injuries expressed as slight changes in hearing may be sufficient to reduce the peripheral drive to higher centres. There may also be memory-related or intrinsic asymmetries in central responses to peripheral input possibly accounting for variability in tinnitus localisation reporting (Cahani et al. 1984; Lee et al. 2021). Tinnitus localisation mechanisms intersect with poorer sound localisation in the model. Further work is needed to extract tinnitus per se, from tinnitus-related hearing deficits, as the causative factor (Hyvärinen et al. 2016). It is difficult to disentangle hearing from tinnitus in terms of the cause or effect of neural change. Even when normal hearing groups are used, the presence of asymmetries outside of the frequencies tested or lesions associated with hidden hearing loss may be drivers for spatial plasticity causing tinnitus.

There is a general consensus that while tinnitus may be acutely triggered by a peripheral injury, central hyperactivity develops over time (Mulders and Robertson 2011). Two main ascending auditory pathways are implicated in creating tinnitus, a medial ascending pathway involving the anterior cingulate cortex proposed to govern motivation and emotion and a lateral ascending perception pathway that includes localisation (De Ridder et al. 2022). The results of several studies collated in this scoping review provide evidence consistent with these pathways. Evidence for lateralisation of tinnitus within the brain is mixed and it may depend on exiting asymmetries in hearing, or somatosensory involvement. Melcher et al. (2000) and Smits et al. (2006) found, in response to sound, IC fMRI activation was abnormally low contralateral to the tinnitus percept in lateralised tinnitus subjects. It is believed that such tinnitus-related activity in the ascending pathways is not selectively filtered and ultimately leads to reorganisation of functional connectivity between and within neural networks responsible for conscious perception, attention, goal-based functioning, and reaction (De Ridder et al. 2022). EEG recordings demonstrated that unilateral and bilateral tinnitus had different resting state oscillation patterns in the auditory cortex, angular gyrus, parahippocampal area and superior premotor cortex (Vanneste Sven et al. 2011b). Left-sided tinnitus resulted in more severe and widespread alterations in white matter in regions associated with relaying sensory information and cognition REF. These results are consistent with a network model of tinnitus (De Ridder et al. 2014). The model has a tinnitus perceptual core of the auditory cortex, prefrontal cortex, and inferior parietal area. The inferior parietal area in particular has a role in spatial perception (Zatorre et al. 2002; Alain et al. 2008). Of all the themes in the model, the mechanisms’ theme studies apply the least rigorous descriptions of tinnitus location. The variability in study findings, particularly in regard to central lateralisation (Folmer 2007), may be attributed to inconsistency in definitions and group allocations. Binary labelling of tinnitus as unilateral or binaural enables easy group allocation and hence recruitment, however just two categories, without very clear exclusion criteria to narrow localisation, is inadequate for study purposes. All current mechanistic studies should be viewed with this significant limitation in mind.

**Figure 2.**
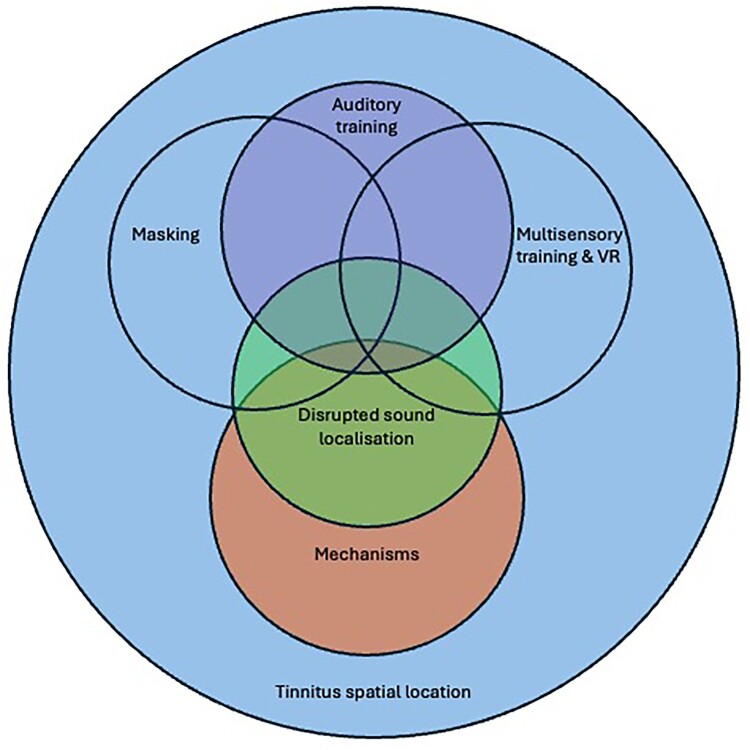
Model of tinnitus spatial localisation derived from the scoping review. Tinnitus perception consists of multiple overlapping themes of: Tinnitus localisation. Effect of tinnitus on localisation. Mechanisms of tinnitus localisation. Masking. Auditory training. Multisensory training & Virtual Reality.

The themes of masking, auditory and multisensory training in the model (top three circles Figure 2) tended to apply more realistic spatial representations of tinnitus. These 3 themes are affected by mechanistic aspects of tinnitus and the general definitions of tinnitus location, but they weren’t compromised by these limitations in terms of meaningful outcomes and confidence in outcomes. This is because to match or cover the tinnitus with sound a reasonable overlap in spatial perception was needed. A systematic review of neurophysiological evidence suggests that tinnitus core regions change with masking (Burton-Harris et al. 2023). Contralateral masking for unilateral tinnitus and monaural masking for bilateral tinnitus has been successful at reducing tinnitus perception (Tyler and Conrad-Armes 1984). The addition of spatial cues to masking sounds improved success in providing tinnitus relief (Searchfield et al. 2016) and distraction from simulated tinnitus (Kubota et al. 2022). It is likely that tinnitus masking is predominately a form of informational masking (Kidd et al. 2008). Informational masking affects the extraction of a signal from neural activity, as opposed to excitatory masking which alters the travelling wave in the cochlea. Spatial similarity contributes to informational masking (Arbogast et al. 2002). The better the spatial match the greater the masking should be. Kubota et al. (2022) did not find spatial overlapping of masker with simulated tinnitus to be the most effective spatial masking, however, simulated tinnitus is known not to be a good substitute for real tinnitus in masking experiments (Tyler and Conrad-Armes 1984). EEG recordings showed the strength of connectivity centred around parietal-occipital regions was changed with effective maskers including spatial information (Durai et al. 2021). Spatial masking may interrupt processing by the dorsal ‘where’ pathway of the dual stream hypothesis (Kubovy and Van Valkenburg 2001).

The brain has the capability to ‘filter out’ parts of the sensory inputs it deems noisy, this possibly fails in tinnitus (Rauschecker et al. 2010). Sound localisation is an important tool for attentional mechanisms that enable focus or distraction from sound (Avan et al. 2015). Spatial attention is implicit in results in which individuals describe their tinnitus as occurring in space, in and around the head. Spatial attention is a combination of bottom-up and top-down influences (Noyce et al. 2023). Bottom-up attention may relate to the unusual and salient aspects of tinnitus, this may be involuntary. Spatial masking may disrupt bottom-up attention to tinnitus by replacing it with a real, less noxious, sound. Top-down attention is under conscious control and equates to individuals focusing on their tinnitus. Attention can also be object-based, focused on streams of tinnitus features (including location) (Durai et al. 2019). Top-down and object-based spatial selective attention to tinnitus may be an important attribute that could be modified in treatments. Several of the studies surveyed reduced tinnitus through active effortful listening to identify objects (Searchfield et al. 2007) or ignoring distractor sounds similar to tinnitus while identifying different target sounds (Wise et al. 2016). These studies were able to demonstrate benefits in reducing tinnitus and improving attention (Searchfield et al. 2007; Wise et al. 2016).

Realistic representation of spatial cues is important for not just tinnitus localisation, but it also appears these may be useful for creating a sense of realty for the tinnitus. Several multisensory training or VR studies were reviewed that reported on the development of spatial audio for tinnitus management (Malinvaud et al. 2016; Park et al. 2022; Draper et al. 2023). VR methods have been applied to tinnitus in other studies but they were not included in the review as they did not use spatial sound, or do not attempt to manipulate tinnitus position (Worms and Deshpande 2021; Deshpande et al. 2022). Modern VR technologies can generate immersive environments. The ability to expose patients to ecologically valid environments with virtual auditory scenes in a safe manner may lead to more effective therapies for the treatment of tinnitus. The novelty of tinnitus perception coupled with its location in peri-personal space would be expected to result in reactions like what would be expected by an unpleasant object in close proximity to us, that is alertness and unease, with attention engaged to understand its source (Ferri et al. 2015). Experience of this perception in a controlled manner through tinnitus location matching may reduce some of the uncertainty and fear about tinnitus. Replication also can help demonstrate the tinnitus experience and so facilitate empathy from friends and family. Use of the spatial information may also potentiate masking, distraction, and selective auditory attention towards real-world sounds. That spatial perception is highly plastic suggests awareness, attention, and reaction to spatially matched tinnitus can be modified by learning. The combination of masking with spatial cues to provide relief followed by effortful training may inhibit the tinnitus perception. Multisensory training and virtual/augmented reality applications may also help to increase the accuracy of, and control over, tinnitus masker locations by leveraging multisensory processes, such as the ventriloquist effect (Witkin et al. 1952). The initial unfamiliarity with tinnitus on first being heard may contribute to the attention it draws (Feldmann 1992). Tinnitus spatial matching (Searchfield et al. 2015) perceptual training (Spiegel et al. 2015; Wise et al. 2016) and VR (Malinvaud et al. 2016; Park et al. 2022; Draper et al. 2023) may facilitate tinnitus adaption as the person with tinnitus can associate it with an external object, and so it no longer continues to monopolise attention. The failure to adapt to tinnitus over time could be related to a disparity in representation across the senses. If tinnitus could be formed from audition and confirmed with a visual, or tactile, avatar, the need for a response may be attenuated. Auditory–visual incongruence may contribute to tinnitus generation, or visual tasks may mediate its perception just as visual tasks may alter pain perception (Don et al. 2017). Auditory, multisensory, and virtual reality training may be effective by facilitating this experience. With experience, it may become easier to inhibit the distraction of tinnitus and focus on real-world sounds (van Moorselaar and Slagter 2020). The one study to include measures of brain activity with VR demonstrated changes in orbitofrontal cortex consistent with improved cognitive control, possibly inhibitory executive control, over tinnitus (Park et al. 2022). A consequence of this training may be an improved ability to mask tinnitus (Searchfield et al. 2007). The combination of training with masking may be synergistic, addressing the multiple parallel pathways and mechanisms creating tinnitus through complementary actions. Training may enable greater relief from tinnitus masking; masking may facilitate greater training benefits. The result may be neural adaptation (Searchfield et al. 2012; Willmore and King 2023). It is recommended clinicians pay attention to the spatial location of their patients’ tinnitus, consider how this may be contributing to their distress, and whether spatial methods can be specifically applied to modify, or resolve, tinnitus perception. Multiple methods targeting the same perceptual process in a harmonised, complementary manner may result in superior clinical outcomes than the use of single therapeutic approaches (Searchfield and Sanders 2022).

This study employed search terms that captured many irrelevant studies. The great majority of rejected studies included reference to neuroanatomical ‘locations’ of tinnitus-related activity, not the process of tinnitus perception in space. Studies of tinnitus associated with SSD were excluded from cataloguing and description. This exclusion was done to prevent an atypical tinnitus population from diluting the focus on spatial perception in the general tinnitus population. The tinnitus data archive (Meikle et al. 2004) indicates that the typical hearing loss for tinnitus clinic patients is a symmetric normal, slopping to mild-moderate loss, very different from SSD. SSD and tinnitus have also been reviewed extensively elsewhere, primarily with regard to the benefits of cochlear implants (Peter et al. 2019). Future reviews of tinnitus spatial perception may wish to employ a systematic review method. However, we believe at this time, a systematic review would be a poor use of resources, given the limited literature, the diversity in methods and research disciplines, and the large proportion of studies with small sample sizes. A meta-analysis would have limited data to review. The relatively small number of studies finally included in this scoping review is surprising, given the importance of spatial perception in our daily lives, but was anticipated by the authors who are engaged in this research field. There, however, has been a growth in research outputs in the last 25 years, most of the articles included in this review were published after the year 2000. Given the relative paucity of research, there are many opportunities for research.

**Figure 3.**
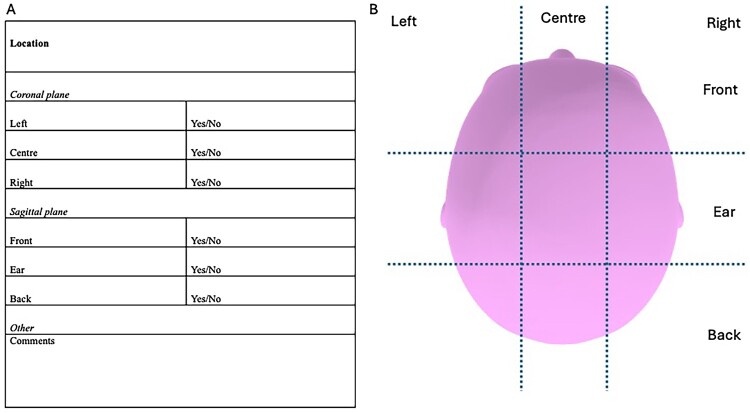
**A,** Recommended minimum reporting for characterisation of tinnitus spatial location. Characterisation could include more than one response. **B,** Visual aid for self-reporting on tinnitus location and clinical chart records.

The greatest limitations of the research reported are the varied and inadequate classification of tinnitus spatial location. We recommend that future localisation focused studies employ improved classification with higher resolution to differentiate between different spatial tinnitus locations. Spatial matching methods are reliable for laboratory and clinical studies (Searchfield et al. 2015a). In circumstances where matching is not possible, e.g. big data capture through surveys, we recommend a simple matrix (Figure 3) that could be easily employed in clinical history taking and self-report. The six categories adequately cover the regions commonly reported in the review and are not burdensome to record (e.g. left-front, centre-ear). Perception of tinnitus outside of the head is infrequent but can be noted using the same matrix with the addition of a comment. Elevation is not included as this is more difficult for individuals to describe, and to keep the tool simple to use. We recommend this matrix as a guideline to standardise reporting of tinnitus location.

## Conclusions

Tinnitus has a definite spatial attribute to its perception. Spatial information can be used to increase patient understanding of tinnitus and its reframing as an auditory object. Spatial sound can be used to improve informational masking and passive distraction. Spatial sounds, and multisensory cues, can be used to reduce selective auditory attention to tinnitus with the goal of improving neural inhibition of an irrelevant distractor. Reporting of the spatial location of tinnitus is inconsistent in the literature. We have recommended minimum standardised reporting method for tinnitus location.

## Supplementary Material

Supplemental Material
