# Supplementary material for: A scoping review of the spatial perception of tinnitus and a guideline for the minimum reporting of tinnitus location
Source: J R Soc N Z. 2024 May 1;55(3):501–19. doi: 10.1080/03036758.2024.2344781 (PMC11841106; doi:10.1080/03036758.2024.2344781)
Supplement: Supplemental Material [file TNZR_A_2344781_SM0966.docx]

**Supplementary**

Table. Outcomes of scoping review summarised (AEP – Auditory Evoked Potential, CBT – Cognitive Behavioural Therapy, EEG – electroencephalography, HRTF – Head Related Transfer Function, THI- Tinnitus Handicap inventory, THQ – Tinnitus Handicap Questionnaire, TFI – Tinnitus Functional Index, MML – Minimum Masking Level, SSD – Single Sided Deafness, VR – Virtual Reality.

| **AUTHORS** | **YEAR** | | **METHOD** | **PARICIPANTS** | **COMMENTS** |  |
| --- | --- | --- | --- | --- | --- | --- |
|  |  |  | |  |  |  |
| Tinnitus localisation | | | | | | |
| Axelsson & Ringdahl | 1989 | Survey random sample | | Human, 337 tinnitus | Tinnitus localised various places in and around the head |  |
| Stouffer & Tyler | 1990 | Survey tinnitus group | | Human, 528 tinnitus | Tinnitus localised various places in and around the head |  |
| Erlandsson et al. | 1992 | Survey clinic | | Human, 163 tinnitus | Three categories only. Unilateral tinnitus localisation was more prevalent in females |  |
| Lim et al. | 2010 | Survey clinic | | Human, 327 tinnitus | Tinnitus localised various places in and around the head |  |
| Bertet et al. | 2013 | Laboratory matching using interaural intensity difference | | Human, 8 tinnitus | Spatial position of avatar merged with tinnitus can be manipulated |  |
| Searchfield et al. | 2015 | Laboratory matching using HRTF | | Human, 34 tinnitus | Matching of 3D location repeatable |  |
| Cuesta & Cobo | 2021 | Survey clinic | | Human, 170 | Three localisation categories only |  |
| Effect of tinnitus on localisation | | | | | | |
| Schmielau et al. | 2009 | Laboratory sound localisation 360 degrees | | Human, 1 tinnitus | Spatial location ability decreased relative to controls |  |
| Niewiarowiczw & Kaczmarek | 2011 | Laboratory sound localisation | | Human, 9 normal hearing 9 hearing loss tinnitus | No difference in localisation for normal and tinnitus participants, but localisation to tinnitus sound poorer |  |
| An et al. | 2012 | Laboratory sound localisation | | Human, 40 with tinnitus 40 no tinnitus | Tinnitus interferes with sound localization ability and that interference was worse for sound originating from the same side as the tinnitus |  |
| Hyvärinen et al. | 2016 | Laboratory sound localisation | | Human, 8 tinnitus, 8 hearing loss, 9 normal hearing | Monaural listening the tinnitus group localized significantly worse with the tinnitus ear |  |
| Liu et al. | 2018 | Laboratory sound localisation | | Human, 26 single-sided deafness with tinnitus 10 normal hearing | SSD subjects with tinnitus performed poorer in sound localization and speech recognition in noise than those without tinnitus |  |
| Elsherif et al. | 2021 | Laboratory sound localisation | | Human, 20 tinnitus 20 normal hearing | Tinnitus diminished sound localization ability |  |
| Long et al. | 2023 | Laboratory sound localisation | | Human, 40 with tinnitus 40 normal hearing | Tinnitus interfered with the ability to localize pure tones |  |
| Long et al. | 2023 | Laboratory sound localisation | | Human, 76 with tinnitus 74 normal hearing | Tinnitus negatively impacted sound source localization |  |
| Mechanisms of tinnitus localisation | | | | | | |
| Heffner & Koay | 2005 | Animal conditioned responses | | Rats, 10 noise exposed | Rats lateralise tinnitus |  |
| Heffner | 2011 | Animal conditioned responses | | Rats, 6 noise exposed, 9 control | Rats lateralise tinnitus |  |
| Arnold et al. | 1996 | PET scans | | Human, 10 with tinnitus | Increase in auditory cortex left for 9, 1 in right |  |
| Lockwood et al. | 1998 | PET scans | | Human, 4 with oral-facial modulated tinnitus | Increase in activity in contralateral auditory cortex |  |
| Mühlnickel et al. | 1998 | MEG | | Human, 10 tinnitus, 15 controls | Tonotopic maps distorted both hemispheres |  |
| Levine | 1999 | Case series | | Human, 6 with somatic tinnitus | Tinnitus ipsilateral to craniocervical injury |  |
| Frank et al. | 2010 | Retrospective evaluation of left temporal rTMS | | Human, 194 | Left-side (ipsilateral) and bilateral tinnitus reduced with left sided TMS |  |
| Jin et al. | 2006 | Animal physiology | | Hamsters, 3 control, 3 (2 days) 3 (8 days) 3 (2 months) post noise | Choline acetyltransferase lateralised to lesioned side |  |
| Chen et al. | 2022 | Magnetic Resonance Imaging | | Human, 19 left-sided 19 right-sided 19 controls | Left-sided tinnitus greater white mater changes |  |
| Cahani et al. | 1984 | Laboratory sound localisation | | Human, 62 with tinnitus | Right-sided tinnitus associated with right-sided hearing loss, left-sided hearing loss tinnitus heard in both ears or louder on the right. |  |
| Meikle & Griest | 1992 | Tinnitus data archive analysis | | Human, 1033 | Left-sided tinnitus greater, may be related to experience shooting guns, but weak association with degree of hearing loss. |  |
| Genitsaridi et al. | 2021 | Secondary data analysis | | Humans, 833 with tinnitus | Hearing asymmetry (maximum interaural difference) most strongly discriminated unilateral from bilateral tinnitus |  |
| Vanneste et al. | 2011 | Laboratory resting state EEG | | Humans, 35 Unilateral 27 bilateral tinnitus | Unilateral and bilateral tinnitus can be differentiated based on their resting state oscillation patterns |  |
| Vanneste et al. | 2011 | Laboratory resting state EEG | | Humans, 46 Unilateral tinnitus, 21 control | Contralateral gamma-band activity in parahippocampus important for lateralisation of tinnitus |  |
| Lanting et al. | 2014 | fMRI | | Humans, 14 Unilateral tinnitus, 16 control | Connectivity not related to lateralisation |  |
|  |  |  | |  |  |  |
| Masking | | | | | | |
| Tyler & Conrad Armes | 1984 | Laboratory masking | | Humans, 10 with tinnitus | Contralateral masking possible |  |
| Johnson & Hughes | 1992 | Laboratory masking | | Humans, 15 with tinnitus | Dichotic (ear ear) and Diotic (heard in head) effectiveness similar Diotic masking requiring higher noise levels |  |
| Tyler & Stouffer | 1992 | Laboratory masking | | Humans, 30 with tinnitus | Masker location in horizontal plan affects level of masking required, correlated binaural maskers most effective |  |
| Searchfield et al. | 2016 | Laboratory masking 1 study, field trials 2 studies | | Humans, study 1 19 with tinnitus, study 2 15 with tinnitus, study 3 10 with tinnitus | User preferences for 3D spatial masking stimulus |  |
| Theodorff et al. | 2014 | Retrospective analysis of a clinical trial | | Human, 89 | Masking and Tinnitus Retraining Therapy more successful when tinnitus localised to the head |  |
| Kubota et al. | 2022 | Laboratory masking 2 studies simulated tinnitus | | Humans, study 1 without tinnitus, study 2 15 without tinnitus | Spatial information reduces the perception level of simulated tinnitus |  |
| Searchfield et al. | 2021 | Field trial case series with EEG | | Human, 11 with tinnitus | Greatest response when masker included spatial cues. Changes in connectivity in the right hemisphere especially at occipitoparietal sites |  |
| Auditory training | | | | | | |
| Searchfield et al. | 2007 | Field trial 15-day auditory object identification and localisation training | | Human, 10 with tinnitus | Reduction in MMLs associated with improved reaction time in attention task |  |
| Wise et al. | 2015 | Field trial 20-day attention and localisation task | | Human, 8 with tinnitus | Significant reductions in THI scores |  |
| Wise et al. | 2016 | Field trial 20-day attention and localisation task with AEPs | | Human, 15 with tinnitus treatment game, 16 with tinnitus control game | Clinically meaningful reduction in TFI for treatment, lasting at least weeks after training, reduction in N1 AEP latency |  |
| Searchfield & Sanders | 2022 | Field trial 90-day digital therapy and masking control | | Human, 31 digital therapeutic, 30 reference app | Treatments therapeutic with spatial masking and attention greater number of clinically meaning TFI change compared to masking app |  |
| Multisensory training & Virtual Reality | | | | | | |
| Spiegel et al. | 2015 | Field trial multisensory training 20 days | | Human, 20 with unilateral tinnitus | Training associated with significant reductions in TFI and rating scale, improved attentional abilities. |  |
| Searchfield et al. | 2021 | Field trial multisensory training 20 days with Fluoxetine, with fMRI | | Human, 18 with unilateral tinnitus | No significant changes in TFI with training, rating scales changed, Fluoxetine did not alter behavioural outcomes of training compared to placebo. Attention neural network ROI changes correlated with significant tinnitus rating changes |  |
| Malinvaud et al. | 2016 | Laboratory trial with follow up VR vs CBT | | Human, tinnitus VR 61, CBT 58, wait list n = 29 | VR immersion allowed voluntarily manipulation of a tinnitus avatar. Results equivalent to CBT. |  |
| Park et al. | 2022 | Laboratory trial with EEG | | Human, 19 with tinnitus | Improvement in THI but not THQ, EEG activity in the orbitofrontal cortex increased |  |
| Bonnet et al. | 2022 | Prism adapation | | Human, case study | Tinnitus moved away from original position following distortion of vision |  |
| Draper et al. | 2023 | Laboratory user experience | | Human, 18 without tinnitus | A beach scene was perceived as the most relaxing |  |
